# Industrial Relevance of Chromosomal Copy Number Variation in Saccharomyces Yeasts

**DOI:** 10.1128/AEM.03206-16

**Published:** 2017-05-17

**Authors:** Arthur R. Gorter de Vries, Jack T. Pronk, Jean-Marc G. Daran

**Affiliations:** Department of Biotechnology, Delft University of Technology, Delft, The Netherlands; USDA Forest Products Laboratory

**Keywords:** aneuploidy, evolutionary adaptation, strain improvement, genome engineering, industrial yeast fermentation, fermentation, industrial yeast

## Abstract

Chromosomal copy number variation (CCNV) plays a key role in evolution and health of eukaryotes. The unicellular yeast Saccharomyces cerevisiae is an important model for studying the generation, physiological impact, and evolutionary significance of CCNV. Fundamental studies of this yeast have contributed to an extensive set of methods for analyzing and introducing CCNV. Moreover, these studies provided insight into the balance between negative and positive impacts of CCNV in evolutionary contexts. A growing body of evidence indicates that CCNV not only frequently occurs in industrial strains of Saccharomyces yeasts but also is a key contributor to the diversity of industrially relevant traits. This notion is further supported by the frequent involvement of CCNV in industrially relevant traits acquired during evolutionary engineering. This review describes recent developments in genome sequencing and genome editing techniques and discusses how these offer opportunities to unravel contributions of CCNV in industrial Saccharomyces strains as well as to rationally engineer yeast chromosomal copy numbers and karyotypes.

## INTRODUCTION

Saccharomyces yeasts are applied in a large and expanding number of industrial processes (1), ranging from traditional applications such as dough leavening (2) and beer (3) and wine fermentation (4) to modern processes such as the production of first- and second-generation fuel ethanol (5, 6), other low-molecular-weight compounds (7), and heterologous proteins (8). Selection and improvement of yeast strains remain essential to meet the complex, diverse, and continually changing performance criteria for industrial applications of Saccharomyces yeasts (9). Improving and extending yeast strain applications can be pursued by exploration of biodiversity, mating, interspecies hybridization, random mutagenesis and selection, evolutionary engineering, targeted genetic modification, or a combination of these approaches (10).

Understanding the genetic basis for industrial performance is invaluable for focusing and accelerating microbial strain improvement. In prokaryotes, genetic variation among related strains and species predominantly encompasses the presence or absence of protein-encoding and regulatory sequences, as well as mutations in these sequences. In eukaryotes, including the Saccharomyces yeasts, differences in ploidy, i.e., variations in copy number of chromosomes, provide an important additional source of genetic diversity (11).

While most eukaryotic cells are euploid, i.e., their chromosomes all have the same copy number, aneuploidy is encountered in nature as well as in manmade contexts. In aneuploid cells, the copy number of one or more chromosomes differs from that of the remainder of the genome. The existence of stable aneuploidy cells implies that chromosomal copy number variation (CCNV) contributes to genetic and physiological diversity within eukaryotic species and, in multicellular eukaryotes, within organisms. The biological significance of CCNV is powerfully illustrated by its impacts on human health. Effects of CCNV of human X and Y chromosomes range from infertility (XXY) to mental retardation (XXXXY), while trisomies of other chromosomes can cause decreased life span, mental retardation, and premature fetal death (12, 13). Spectacular CCNV occurs in most human cancer cell lines, leading to chromosome numbers of up to 90, and has been linked to the cancer hallmark of increased genome instability (14). Targeting of aneuploid cells is therefore considered a potential strategy for cancer therapy (15). Use of polyploid plants and animals in agriculture is related to their increased size and infertility (16, 17), while allopolyploid plants additionally combine industrially relevant traits from two parental genomes (18, 19). As will be discussed in this paper, CCNV is also an important phenomenon in industrial strains of Saccharomyces yeasts, whose history often involves prolonged domestication and/or industrial strain improvement.

Saccharomyces cerevisiae is an important model for studying how aneuploidy arises during mitotic and meiotic cell division, how it affects growth, and how it influences evolution of eukaryotes. These research fields are discussed in recent specialized review papers (20–22). The present paper specifically aims to review current knowledge on the analysis, occurrence, and significance of CCNV in Saccharomyces yeasts in industrial contexts. To this end, we review methods for analyzing CCNV in yeast strains, the mechanisms by which CCNV can arise spontaneously or be induced in the laboratory, and the mechanisms by which CCNV can negatively affect fitness of yeast cells. Subsequently, we discuss the occurrence and significance of CCNV for domestication and development of industrial strains of Saccharomyces yeasts and its relevance in evolutionary engineering.

## METHODS FOR CCNV ANALYSIS IN YEASTS

**FIG 1:**
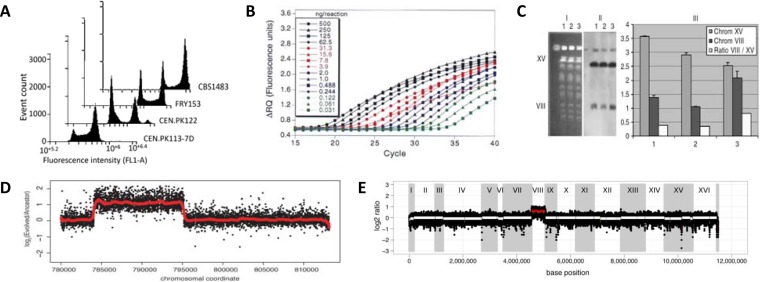
Methods to analyze chromosome copy number and DNA content in yeast cells. (A) Absolute quantification of the DNA content of strain CBS1483 by flow cytometry using the DNA-intercalating dye Sytox Green and calibration with three strains of known ploidy. (Adapted from reference 23.) (B) qPCR fluorescence profiles for different initial concentrations of a template DNA sequence can be used to infer the amount of initial template in a reaction and to calculate relative copy numbers of different parts of the template DNA. (Republished from reference 157.) (C) Chromosome copy number determination of S. cerevisiae variants using contour-clamped homogeneous electric field electrophoresis and Southern blotting. I, stained CHEF gel; II, Southern blot hybridization; III, quantification of the hybridization bands. Lanes 1 and 2 show two disomic knockout strains that have only a single copy of chromosome VIII, while lane 3 shows a diploid control strain. (Modified from reference 28 with permission [copyright 2005 John Wiley & Sons Ltd.].) (D) Copy number estimation of chromosome II by array comparative genomic hybridization of an evolved strain relative to its unevolved parental strain. Deviating copy number can be detected by significant deviations of the measured signal and has been accentuated by a red line. (Republished from reference 108.) (E) Copy number estimation of the genome of the wine production strain VL3, based on whole-genome sequencing and read depth analysis. A marked increase of the read depth for chromosome VIII indicates a gain of copy of that chromosome. (Adapted from reference 117.)

Analysis of chromosomal copy numbers in yeasts predominantly relies on five, largely complementary methods (Fig. 1). Flow cytometry analysis of cells stained with fluorescent DNA-intercalating dyes, using reference strains for calibration, enables absolute quantification of cellular DNA content and overall ploidy (23). The choice of fluorescent dyes should consider excitation/emission spectra, RNA/DNA specificity, mutagenicity, effects on viability, and the required accuracy (24). When the fluorescent dye does not compromise viability, fluorescence-activated cell sorting (FACS) can be used to select cells with a deviating DNA content. FACS-based selection has enabled selection of mutants whose DNA content differed by less than 2% from that of the parent population (25). While this FACS approach cannot select cells with specific chromosome amplifications or deletions, it can preselect cells with a deviating overall DNA content.

Contour-clamped homogeneous electric field (CHEF) electrophoresis separates yeast chromosomes on agarose gels and is used to analyze chromosome complements (karyotypes) of yeast strains (26, 27). Southern hybridization of CHEF gels can reveal copy numbers of individual chromosomes by comparison of hybridization intensity with reference strains (Fig. 1C) (28). However, the accuracy of CCNV estimates obtained by this method is limited.

Copy numbers of individual yeast chromosomes can be analyzed by quantitative real-time PCR (qPCR, Fig. 1B), using primers that amplify chromosome-specific genomic sequences (29). Accuracy of PCR-based copy number estimates can be boosted by digital droplet PCR (ddPCR), which uses microfluidics to generate thousands of replicate PCRs in water-in-oil emulsions (30, 31). Since qPCR analysis estimates copy numbers of only the amplified region(s), additional methods are required to assess whether these reflect copy number variations of entire chromosomes or of specific chromosomal regions (segmental aneuploidy).

Array comparative genomic hybridization (aCGH) compares local copy number differences by hybridizing genomic DNA from related yeast strains to oligonucleotide arrays (Fig. 1D) (32). Depending on oligonucleotide size and genome coverage of the arrays, copy number variations can be analyzed across entire genomes at resolutions down to 20 bp (33).

High-resolution, accurate analysis of CCNV in yeast increasingly depends on next-generation sequencing (NGS) of entire yeast genomes (34). NGS enables ploidy estimation from allele frequency in the whole genome and in specific regions (35). Moreover, when sequence bias in DNA isolation and/or sequencing (36) is prevented, the number of reads generated for any particular sequence (i.e., its read depth) directly reflects its copy number relative to the remainder of the genome (Fig. 1E) (37). Computational tools assist CCNV identification via read depth, either by mapping of NGS reads to a preassembled genome sequence or via *de novo* genome assembly (38). With both approaches, the accuracy of copy number estimates increases with increasing sequencing coverage. When many copies of a chromosome are present in a yeast strain, (dis)appearance of a single copy causes only a small relative change. Accurate analysis of aneuploid yeast genomes with large variations in chromosomal copy numbers therefore requires high sequencing coverage. Short-read-length NGS methods currently provide the most cost-effective access to high sequencing depth (>100× coverage at read lengths from 75 to 400 bp can be obtained routinely with, for example Illumina and Ion Torrent platforms). Sequencing reads can be mapped to a preassembled, accurate reference genome similar to that of the sequenced strain, yielding accurate CCNV estimates. If no such reference genome is available, *de novo* assembly of the genome and subsequent copy number analysis can provide unbiased and more accurate results (23). However, short-read-length NGS does not allow assembly of repetitive regions whose length exceeds the read length, such as TY, subtelomeres, and ribosomal DNA (rDNA) sequences in Saccharomyces genomes. *De novo* genome assembly is strongly facilitated by long-read-length sequencing platforms (e.g., Pacific Biosystems and Oxford Nanopore Technologies), either alone or combined with short-read-length data. Moreover, when genes are present in multiple nonidentical copies, it can be difficult to perform full reconstruction of duplicated alleles (“phasing”) (39). Indeed, when two single nucleotide polymorphisms (SNPs) occur in only one copy of a gene, nucleotides can be assigned to a specific allele only if individual reads that cover both variable positions are available. Allelic reconstruction, and by extension reconstruction of (parts of) chromosome copies, is enhanced by the use of long-read or mate-pair sequencing data (39). Long-read sequencing technologies still have higher error rates than short-read platforms. Fast developments in real-time, single-molecule methods for replication (Pacific Biosystems) or nanopore (Oxford Nanopore Technologies) sequencing enable generation of extremely long reads with increasing accuracy (40–43) and are likely to transform whole-genome resequencing (44). The potential of long-read sequencing to capture entire chromosome arms or even entire chromosomes within a single read offers unique possibilities to unravel chromosome structure, translocation breakpoints, and allelic variation among duplicate chromosomes and chromosomal fragments (41).

## INDUCTION OF CHROMOSOME MISSEGREGATION

**FIG 2:**
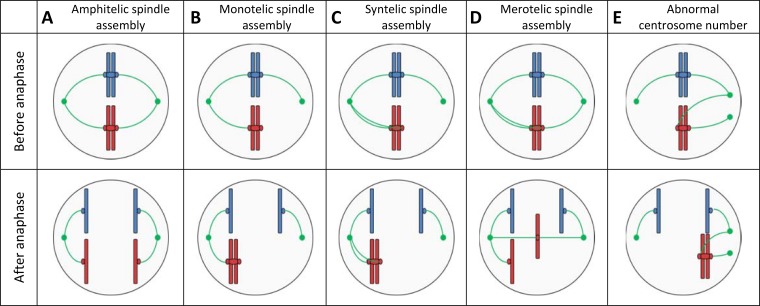
Schematic representation of chromosome segregation and of the common mechanisms leading to chromosome missegregation. Two chromatids of two different chromosomes are shown in red and blue, with their centromeres and kinetochores. In green, the centrosomes are shown with the assembled microtubule attached to the kinetochores of the chromatids. For each case, the microtubule-kinetochore assembly is shown before and after the anaphase. (A) Correct chromosome segregation is achieved by amphitelic spindle assembly, where microtubules connect each chromatid to a different centrosome, resulting in separation to opposite cellular poles during anaphase and maintaining a stable karyotype in the daughter cells (45). (B and C) If only one of the chromatids is attached to a centrosome or both chromatids are attached to the same kinetochore, referred to as monotely and syntely, respectively, proceeding to anaphase would result in the missegregation of both chromatids to that centrosome. However, monotelic and syntelic spindle assemblies are detected at the spindle assembly checkpoint and therefore rarely cause chromosome missegregation. (D) In the case of a merotelic spindle assembly, a chromatid is attached to both centrosomes and, as a result, cannot migrate to a cellular pole. The resulting random segregation of the lagging chromosome can cause missegregation, damage, and micronucleus formation (158). (E) When more than two centrosomes are formed, random attachment of chromatids can result in chromosome missegregation due to chromosome lagging or unequal chromosome segregation (159).

The anaphase of the eukaryotic cell cycle has evolved to conserve chromosomal copy number during cell division. Its crucial steps include chromatid cohesion, centrosome formation at opposite cell poles, kinetochore-microtubule attachment, and quality control at the spindle assembly checkpoint (45). Imperfections in any of these steps can cause chromosome missegregation and, thereby, CCNV in eukaryotic cell populations, tissues, and tumors (45–47). Even in cell lines without predisposing defects, chromosome missegregation occurs, albeit at very low frequencies (21, 48). In yeast, chromosome missegregation can occur during mitosis (48) and, with a higher incidence, during the meiotic process of sporulation (49). Figure 2 provides a schematic overview of mechanisms by which missegregation of chromosomes can occur.

A wide range of chemical and physical stress factors increase the incidence of chromosome missegregation in growing cultures. Stimuli that increase occurrence of CCNV in mitotic yeast cultures include nutrient-limited growth (50), heat shock (51), UV or X-ray irradiation (52), and chemical stress. Chemical compounds such as nocodazole, fumaronitrile, and methyl benzimidazole-2-yl-carbamate induce a high incidence of chromosome missegregation in S. cerevisiae (53–55). Polar aprotic solvents, including ethanol esters, are other known inducers of CCNV (56), and high concentrations of ethanol itself have also been reported to enhance chromosome missegregation in fungal cells (57). Exposure to high ethanol concentrations may therefore contribute to the frequent occurrence of CCNV in industrial yeast strains used for production of alcoholic beverages and fuel ethanol (see below).

Chromosome missegregation can also be stimulated by genetic factors. Increased ploidy strongly enhances chromosome missegregation (58), in particular when uneven numbers of chromosome sets preclude equal distribution of chromosomes during meiosis (59). Strongly increased chromosome missegregation rates have also been observed in allopolyploid Saccharomyces yeasts, which carry chromosomes from different parental species and show a high incidence of aneuploidy (60). Since aneuploidy itself, including segmental aneuploidy, also stimulates chromosome missegregation, aneuploid cells are more prone to acquire further CCNV (61).

In contrast to chemical, physical, and genetic stresses, which affect segregation of all chromosomes, targeted molecular genetic approaches enable elimination or amplification of specific chromosomes. In S. cerevisiae, copy gain and loss of specific chromosomes have been achieved by cloning a strong inducible promoter upstream of the centromere of the targeted chromosome (62, 63). When induced, transcription from the promoter interferes with centromere function, thus causing missegregation during mitosis. Aneuploid daughter cells that have lost or gained a copy of the targeted chromosome can then be isolated from the resulting culture. Alternatively, by crossing with *kar1* null mutants, mating is prematurely aborted but chromosome transfer between nuclei can still occur, yielding aneuploid cells. Aneuploidy of specific chromosomes can be easily selected for when they carry marker sequences (64).

## NEGATIVE IMPACTS OF CCNV ON FITNESS

Aneuploid yeasts typically show a reduced fitness relative to congenic euploid strains (64). The molecular basis of generic transcriptional responses to aneuploidy remains to be fully elucidated. Reported transcriptional responses in aneuploid strains include downregulation of genes involved in cell growth and proliferation and upregulation of genes involved in the environmental stress response (ESR) (64, 65). Studies on the impact of gain or loss of chromosomes in otherwise euploid yeast strains showed that the aneuploidy-associated stress response (AASR) includes increased genome instability, low sporulation efficiency, reduced growth rate, increased nutrient uptake rates, and reduced replicative life span (21, 66, 67). Phenotypic consequences of chromosome gain and those of chromosome loss are similar, suggesting that the responsible cellular mechanisms overlap (68). AASR intensity is positively correlated with the length of the affected chromosome(s) and with the number of affected genes (20, 64, 69). A much less pronounced AASR in polyploid strains has been attributed to a smaller relative impact on chromosome number (64, 70). The absence of AASR-related phenotypes upon introduction of a yeast artificial chromosome harboring nontranscribed mammalian genes indicates that AASR is not due to increased DNA content *per se* (64).

Genome instability of aneuploid yeasts has been linked to the missegregation events that cause aneuploidy and, in particular, to “lagging” (Fig. 2D) of chromosomes during anaphase. DNA damage and imperfect repair of lagging chromosomes cause mutations, deletions, and translocations (71, 72). Additionally, formation of transient micronuclei by lagging chromosomes increases the mutation rate during subsequent mitosis (73, 74). At a longer time scale, aneuploidy promotes generation of CCNV by enhancing chromosome missegregation and mitotic recombination as well as by impairing DNA repair (61, 75, 76). Impaired sporulation of aneuploid strains has been linked to disruption of homologous chromosome pairing during meiosis (77). AASR-related cell cycle defects involve slow accumulation of G_1_ cyclins, causing an abnormal delay in the G_1_ phase (78, 79).

CCNV-associated changes in gene dosage can directly affect expression levels of the affected genes. Typically, gain or loss of a chromosome coincides with an increased or decreased expression level, respectively, of the large majority of expressed genes that it carries (80). Correct subunit folding and assembly of multiprotein complexes (29, 64, 81, 82), which strongly depend on subunit stoichiometry (83), can be disturbed when one or more subunits are encoded by aneuploid chromosomes. A resulting “overload” of the cellular protein folding machinery can cause accumulation of un- and misfolded proteins and proteotoxic stress (67, 70). Indeed, some aneuploid strains show increased sensitivity to inhibitors of protein folding and degradation (84) and impaired functionality of the proteasome, the chaperone Hsp90, or endocytosis-mediated protein degradation (66, 70). Energy costs of protein misfolding and protein overproduction have been implicated in the increased nutrient consumption and slow growth of aneuploid yeast strains (85). The correlation between protein level and gene copy number is not always straightforward (29, 64), and situations have even been described in which the transcript level of individual genes decreased with increasing copy number (86–88). Signaling cascades and transcriptional regulation are among the core cellular systems that can be affected by aneuploidy (89). The impact of gene-dosage-related changes in gene expression on AASR (29) can be further intensified or attenuated by mutations in genes on nonaneuploid chromosomes (90). Such in *trans* effects can, for example, be related to stoichiometric imbalances in protein complexes or pathways, unspecific protein interactions, protein folding, and degradation (81).

Sensitivity to AASR is yeast strain dependent (91, 92). In tolerant strains, mutations that attenuate AASR, such as a loss-of-function mutation in the deubiquitinating enzyme Ubp6p, were identified (82). While not all mutations involved in AASR tolerance are known, its relevance is amply demonstrated by the frequent occurrence of aneuploidy in wild, clinical, and industrial isolates of Saccharomyces yeasts (35, 91, 93).

## CCNV IN EVOLUTIONARY ENGINEERING

In addition to negative impacts on cellular fitness, chromosome-specific effects of CCNV can also confer fitness benefits in specific environmental or genetic contexts. Indeed, CCNV offers a fast way to modify gene copy number during natural evolution of eukaryotes and to increase evolvability by allowing neofunctionalization of amplified essential genes (51, 94–96). Under selective conditions, mutants with CCNV will outgrow the parental population whenever positive effects of CCNV on fitness outweigh any negative impacts of AASR, while further mutations that enhance positive effects or decrease AASR can further increase the initial fitness benefit. CCNV is therefore seen as a significant contributor to evolutionary adaptation in eukaryotes (51, 97).

Technically, adaptive laboratory evolution (ALE) encompasses prolonged cultivation of microorganisms under defined conditions, combined with an analysis of the phenotypic and/or genotypic changes that occur during evolutionary adaptation (98). ALE approaches that are specifically designed to select for industrially relevant traits are often referred to as evolutionary engineering (99, 100). Resequencing of evolved strains can provide insight into the genetic basis for industrially relevant traits and enable its reverse engineering into naive, nonevolved strains (101). Evolutionary engineering is particularly attractive for food and beverage applications, since it does not involve recombinant DNA techniques and associated consumer acceptance and regulatory issues (102).

While, on the time scales involved in natural evolution and speciation, CCNV is considered to be a transient adaptation mechanism that is usually replaced by more elegant and efficient mutations (103, 104), most ALE experiments with yeasts cover only 50 to 500 generations of selective growth. It is therefore not surprising that CCNV is frequently encountered during ALE of Saccharomyces yeasts, for example, for the selection of suppressor mutants (Table 1). Numerous evolutionary engineering studies have linked CCNV to industrially relevant traits, ranging from tolerance to products or inhibitors to improved kinetics of sugar fermentation or sedimentation behavior of yeast cultures (Table 1). In some cases, ALE even resulted in complete duplication of the genome of haploid S. cerevisiae strains, for instance, after selection for glucose-limited growth, high ethanol tolerance, and increased sedimentation (105–107). In the last case, increased ploidy played a major role in shaping an evolved, multicellular phenotype.

**TABLE 1:** Examples of whole-chromosome copy number variations acquired during laboratory evolution experiments with Saccharomyces cerevisiae strains^*a*^

Selected phenotype	Aneuploid chromosome(s)	Confirmed causality	Contributing gene(s)	Reference
Biomass sedimentation	Whole-genome duplication	Yes	*ACE2*	105
Glucose-limited growth	Whole-genome duplication	Yes		107
High temp tolerance	III (+1)	Yes	17 individual genes	103
High pH tolerance	V (+1)	Yes		103
Glucose-limited growth	I (+1), III (+1), V (+1)	No		108
Phosphate-limited growth	IV (+1), VI (+1), X (+1), XIII (+2), XVI (+1)	No		108
Lactate utilization by *jen1*Δ strain	III (+1)	Yes	*ADY2*	112
Xylose utilization	I (−1)	No		160
*p*-Coumaric and ferulic acid tolerance	XIV (+1)	No		160
Copper tolerance	II (+1), VIII (+1)	No	*CUP1*, *SCO1*, and *SCO2*	104
Galactose tolerance	VIII (+1)	Yes	*GAL80*	161
Ethanol tolerance	III (+1), VIII (+1)	No		106
Radicicol resistance	XV (+1)	Yes	*STI1* and *PDR5*	113
Fluconazole resistance	VIII (+1)	No	*ERG11*	113
Tunicamycin resistance	XVI (−1)	Yes		113
Benomyl resistance	XII (−1)	No		113
Suppressors of *MEC1* deficiency	IV (+1)	Yes	*RNR1*	162
Suppressors of *MYO1* deletion	XIII (+1), XVI (+1)	Yes	*HSP82*, *HSC82*, *RLM1*, and *MKK2*	94
Suppressors of *RPS24A* and *RNR1* deletion	IX (+1)	No	*RPS24B* and *RNR3*	163
Suppressors of telomerase insufficiency	VIII (−1)	No	*PRP8*, *UTP9*, *KOG1*, and *SCH9*	164

a In the examples listed, the acquired CCNV was hypothesized to contribute to the selected phenotype. “Confirmed causality” indicates that a causal link between CCNV and the phenotype acquired during laboratory evolution was experimentally confirmed. In cases where the impact of a CCNV on phenotype was linked to specific genes, this is also indicated. Segmental aneuploidies observed in the cited studies are not included in the table.

In addition to whole-chromosome copy number variations, ALE frequently involves segmental aneuploidies (108–111). While both can be identified by analysis of high-coverage, short-read NGS data, precise definition of duplication and/or translocation events and karyotypes involved in segmental aneuploidy generally requires additional analysis by long-read sequencing or diagnostic PCR (110, 111).

Several methods can be applied to test if segmental or whole-chromosome aneuploidies do indeed contribute to phenotypes acquired in an ALE experiment. In some cases, hypothesis-based deletion or amplification of one or more genes on (an) affected chromosome(s) can directly confirm the relevance of a CCNV. For example, an increased copy number of chromosome III in *jen1*Δ mutants evolved for restoration of lactate transport could be rapidly linked to the *ADY2* monocarboxylate-transporter gene on this chromosome (112). Overexpression or deletion studies were also successfully used to identify 17 genes that contributed to the benefit of a copy gain of chromosome III in an S. cerevisiae strain evolved for heat tolerance (103). Alternatively, the relevance of a CCNV in an evolved strain can be tested by introducing the deviating chromosome copy number in a euploid strain, e.g., via transcriptional interference with centromere function (103, 113). Similarly, the chromosome copy number variation can be reverted to wild type, e.g., by sporulation and analysis of segregants with wild-type karyotypes (103, 113). Although the method is not routinely applied, specific chromosomal regions that contribute to an acquired phenotype can be identified by targeted introduction of segmental aneuploidy of sets of tiled chromosomal regions (114). Two recently described PCR-based methods enable duplication or deletion of chromosome segment copies by introduction of telomere seed sequences and of an additional centromere to generate an additional autonomously replicating chromosome fragment. By introducing centromere and telomere seed sequences pointing outward of the region of interest, this region will be duplicated on an additional, independently replicating chromosome (115). Conversely, by introducing a centromere and telomere seed sequences pointing into the region of interest, the targeted chromosome is split into two autonomously replicating chromosomes that no longer contain the targeted region (116). This approach enables a nonbiased, systematic analysis of the positive and negative contributions of chromosomal regions and/or individual genes.

## CCNV IN INDUSTRIAL SACCHAROMYCES YEASTS

Aneuploidy has been observed in Saccharomyces strains used in diverse industrial applications, including dough leavening, bioethanol production, beer brewing, spirit production, wine fermentation, and production of cacao and coffee (Table 2). In industrial strains, CCNV may have occurred during centuries-long domestication processes and/or during strain improvement programs that involved CCNV-inducing mutagenesis procedures such as UV irradiation (52).

**TABLE 2:** Examples of CCNV in industrial Saccharomyces strains^*a*^

Strain	Species	Industrial product	Approximate overall ploidy	Aneuploid chromosome(s)	Reference
BR001	S. cerevisiae	Bread	4n	IX (+1)	93
BR004	S. cerevisiae	Bread	4n	IX (+1)	93
E-IM3	S. cerevisiae	Cacao	3n	VII	165
AY529517	S. cerevisiae	Cacao	2n	IV, XII	165
YE 2-2	S. cerevisiae	Coffee	3n	I, XV, XVI	165
JV2	S. cerevisiae	Coffee	4n	Extensive aneuploidy	165
Y-393	S. cerevisiae	Kefir	3n	I, III, IX	165
YJM1356	S. cerevisiae	Cider	2n	I (+2)	147
YJM1439	S. cerevisiae	Ginger beer	2n	VIII (+2)	147
FostersO	S. cerevisiae	Ale beer	>2n	III (+1), XIV (−1)	117
FostersB	S. cerevisiae	Ale beer	>2n	III (+1), V (+1), XV (+1)	117
CBS1483	S. cerevisiae × eubayanus	Lager beer	>2n	Extensive aneuploidy	23
CBS1270	S. cerevisiae × eubayanus	Lager beer	>2n	Extensive aneuploidy	23
AWRI796	S. cerevisiae	Wine	2n	I (+1)	117
VL3	S. cerevisiae	Wine	2n	VIII (+1)	117
F-12	S. cerevisiae	Flor wine	2n	VII (+1), XIII (+2)	130
SA001	S. cerevisiae	Sake	2–3n	V (+1)	93
SA003	S. cerevisiae	Sake	2–3n	I (+1)	93
SP011	S. cerevisiae	Spirits	2n	I (−1), III (−1), VI (−1), IX (−1), XII (−1)	93
SP001	S. cerevisiae	Spirits	2n	I (−1), VI (−1)	93
Y-999	S. cerevisiae	Bioethanol from starch	3n	III	165
CBS7960	S. cerevisiae	Bioethanol from sugarcane	2n	VIII	165
ZTW1	S. cerevisiae	Bioethanol from corn mash	3n	IX (+1)	166

a The overall ploidy of the strains and identified aneuploid chromosomes are indicated. For strains in which the copy number deviation from euploidy has been determined, this is reported between parentheses. Extensive aneuploidy refers to strains with more than 10 aneuploid chromosomes. Segmental aneuploidies that occur in many of these strains are not indicated in the table.

Currently available information suggests that aneuploidy is not prevalent among S. cerevisiae strains used in dough leavening, bioethanol production, ale-type beer fermentation, and distilled-beverage production. In these strains, aneuploidy typically involves small deviations in copy number of one or a few chromosomes (117–119). Since accurate information is available for only a few of the many hundreds of such strains stored in culture collections, the incidence of CCNV may well be underestimated. Indeed, a recent whole-genome sequencing study revealed extensive CCNV among several beer-related S. cerevisiae strains that were previously assumed to be mostly euploid (93).

There is ample evidence that copy numbers of individual genes or loci affect industrially relevant traits of S. cerevisiae strains. For example, rates of sucrose, maltose, and melibiose fermentation correlate with copy numbers of *SUC*, *MAL*, and *MEL* loci, respectively (120–122), while proline utilization rates correlate with the copy number of the *PUT1* proline oxidase gene (123). So far, the industrial significance of CCNV in industrial S. cerevisiae strains has not been systematically explored. S. cerevisiae ZTW1, a strain isolated from corn mash used in a Chinese bioethanol factory, provides an interesting exception. In this strain, chromosomal and segmental aneuploidy were shown to directly contribute to industrially relevant traits, including copper tolerance and ethanol yield (124).

Consistent with the increased rate of chromosome missegregation in alloploid cells, aneuploidy is highly prevalent among wine and lager-type beer yeasts originating from domestication of natural hybrids of different Saccharomyces species. Despite its frequent occurrence, the impacts of aneuploidy in these genetic contexts have not been explored in depth, and it is unclear how AASR and chromosome-specific copy number effects compare to those observed in otherwise euploid S. cerevisiae strains. In general, these alloploid genomes tolerate aneuploidy well, with massive diversity in chromosome copy numbers across strains (23, 125, 126). Some aneuploid lager brewing yeasts even sporulate, albeit at low efficiency, by anomalous cell division (79). Wine yeasts include S. cerevisiae × Saccharomyces kudriavzevii, S. cerevisiae × Saccharomyces uvarum, and S. cerevisiae × S. kudriavzevii × S. uvarum hybrids (127, 128), many of which are alloaneuploids, with a large diversity in chromosome copy numbers (129). Aneuploidy has a strong impact on performance of “flor” wine yeast. An increased copy number of chromosome VII, which carries the alcohol dehydrogenase genes *ADH2* and *ADH3*, correlated with increased ethanol oxidation capacity of the characteristic vellum formed by these yeasts during sherry wine fermentation (130).

Saccharomyces pastorianus lager beer brewing strains have long been assumed to originate from a hybridization event involving S. cerevisiae and another Saccharomyces species (131). The genome of the cold-tolerant species Saccharomyces eubayanus, first isolated in Patagonia in 2011 (132) and later also found in North America, Asia, and New Zealand (132–135), was shown to exhibit a 99.56% identity with the non-cerevisiae part of S. pastorianus genomes (136). It is postulated that, after one or more spontaneous hybridization events, centuries of domestication and selection of the resulting S. cerevisiae × S. eubayanus hybrid(s) in brewing environments generated the current diversity of lager brewing strains (137, 138). S. cerevisiae × S. eubayanus hybrids made in the laboratory combine at least two important brewing-related characteristics of their parents. The S. cerevisiae subgenome contributes the ability to ferment maltotriose, a major fermentable sugar in wort, while low-temperature performance, essential for the lager brewing process, is conferred by the S. eubayanus subgenome (139, 140).

Historically and mainly based on geographical origin, two groups of S. pastorianus strains were distinguished. Group I (Saaz-type) strains tend to ferment well at low temperatures but generally show poor maltotriose fermentation. Conversely, group II strains (Frohberg type) tend to have higher optimal growth temperatures and ferment maltotriose well (141). These phenotypic differences correlate with ploidy and with the contribution of genetic material from the two subgenomes. Consistent with their better performance at low temperature, group I strains contain more S. eubayanus DNA, while some S. cerevisiae chromosomes can even be absent (e.g., S. cerevisiae chromosome III is absent in all group I strains sequenced so far) (23, 32, 141–143); group II strains generally have a more balanced genome composition, with (multiple) chromosomes from both S. eubayanus and S. cerevisiae (23, 32, 141–143). These differences have been proposed to reflect different hybridization histories of the two groups (144). In this model, group I derives from an original hybridization event involving a haploid S. cerevisiae strain and a haploid or diploid S. eubayanus strain, while group II strains arose from hybridization of a diploid S. cerevisiae strain with a haploid S. eubayanus strain (23) or from two subsequent hybridization events (141). Different hybridization histories appear to be contradicted by conserved chromosome rearrangement breakpoints in group I and group II strains (32, 143). However, these might also have evolved independently due to fragility of the breakpoint and/or by conferring a selective advantage (145). The latter hypothesis is consistent with ALE studies with an S. uvarum × S. cerevisiae hybrid in nitrogen-limited cultures, which selected for recombination between alloploid chromosomes in the *MEP2* ammonium permease gene (146).

Two key brewing-related properties of S. pastorianus strains have been correlated with CCNV. Production of diacetyl, an important off-flavor in lager beers that needs to be removed at the end of fermentation (“Ruh” phase), correlated with copy number of chromosomes III, VIII, X, XII, and XIV (23). These chromosomes harbor genes involved in the valine biosynthesis pathway, which generates α-acetolactate, the precursor for diacetyl production. Similarly, Ca^2+^-dependent flocculation, which is essential for yeast sedimentation during brewing, positively correlated with copy numbers of chromosomes I, VI, XI, and XII, all of which harbor flocculin genes (23).

## OUTLOOK: UNDERSTANDING AND ENGINEERING CCNV IN INDUSTRIAL CONTEXTS

Whole-genome sequences of environmental and industrial isolates of Saccharomyces species, which are becoming available at a rapid and still accelerating pace (35, 93, 147), confirm the relevance of CCNV for the natural diversity, domestication, and industrial strain improvement of these yeasts. Experimental hybridization of strains from different Saccharomyces species is rapidly gaining popularity as a strategy for strain improvement and product diversification of wine and beer yeasts (139, 148, 149). Traits that have been improved by hybridization include fermentative vigor over wide temperature ranges and concentrations of minor fermentation products (150), flocculation capacity (151), and sugar uptake kinetics (152). Moreover, ploidy of laboratory-made hybrid strains correlates with fermentation rates, ethanol yield, and concentrations of aromatic esters (148). In view of the higher tendency of alloploid and allopolyploid genomes to develop aneuploidy, CCNV is likely to be a key factor in the stability and further diversification of the resulting strains.

Targeted introduction of CCNV, e.g., by using drugs that interfere with chromosome segregation, is rarely applied in industrial strain improvement (10). Use of the mitotic inhibitor methyl benzimidazole-2-yl-carbamate (MBC) to mutagenize the aneuploid bioethanol strain ZTW1 demonstrates the potential of this approach (153). Treatment of strain ZTW1 with MBC yielded strains with an improved fermentative capacity under industrial high-gravity conditions (119), enhanced viability after drying (154), and higher final ethanol titer (124). These observations and the frequent appearance of CCNV in ALE suggest that such interference with chromosome segregation may deserve reconsideration in industrial yeast strain improvement.

The relatively small number of cases in which molecular mechanisms by which CCNV contributes to industrial performance of Saccharomyces yeasts have been investigated in detail often identified gene dosage effects as a key contributor. Allelic variation of amplified genes can be an additional, as-yet-underexplored source of industrially relevant diversity within strains that carry CCNV, especially in alloploid strains with a long history of domestication and/or strain improvement. Novel long-read DNA-sequencing approaches (e.g., nanopore MinION sequencing [41]) should enable a much faster identification of such allelic variations and of their correlation with industrially relevant traits, including subtle differences in flavor and aroma production. Recent developments in genome editing, including the advent of CRISPR (clustered regularly interspaced short palindromic repeat)-based techniques (155, 156) and methods for experimentally introducing defined, segmental aneuploidies (115, 116), will accelerate the functional analysis of CCNV. Moreover, these techniques will enable rapid introduction of relevant mutations into strains that do not contain CCNV, without the potential disadvantages of AASR. The combination of these developments will enable a more thorough investigation of the importance of CCNV for the performance of industrial strains and is likely to open the way to using CCNV induction as a tool for strain improvement, either by direct generation of improved strains or by identification of chromosome fragments or genes whose copy number affects industrial performance.
